# 

**Published:** 1902-06

**Authors:** 


### A. V. M. A.

### MINNEAPOLIS, MINN., SEPTEMBER, 1902.

THE local committee of arrangements for the next meeting of the A. V. M. A. has been appointed, and has organized upon a somewhat new plan. A large committee was appointed, and this has been divided into several subcommittees, each one being assigned a certain portion of the work, their plans and methods to be approved by the general committee. There is going to be a great meeting in Minneapolis this fall. Don't forget to be there when the first gavel falls.

Dr. C. J. Marshall, State Secretary for Pennsylvania, is addressing the entire State Association membership and many other veterinarians of the State in the interests of the Minneapolis meeting.

### ASSOCIATION MEETINGS.

The next regular meeting of the Veterinary Medical Association of New Jersey will be held at Stetter's Assembly Hall, 842 Broad street, Newark, N. J., July 10, 1902. Meeting to be called at 10 A. M. An interesting programme is being arranged.

### PERSONALS.

Dr. James W. Sallade, of Schuylkill Haven, Pa., who has lately been appointed Steward of Schuylkill County, met with a painful accident in April from a kicking, vicious mare fracturing a limb and bruising him so badly as to confine him to his bed for several weeks.

Dr. D. E. Salmon, Chief of the Bureau of Animal Industry, was the guest of the Philadelphia Pathological Society on Thursday evening, April 25, 1902.

Dr. W. L. Rhoads, of Lansdowne, Pa., is known among his friends as a fighter. He has just fought a horse case to a successful issue.

Dr. N. A. Christianson, formerly of Magnolia, Minn., has removed to Luverne, in the same State.

Dr. R. G. Rice, of Towanda, Pa., is secretary of the town Board of Health.

Dr. I. K. Atherton, of the Bureau of Animal Industry, has been transferred from Marshalltown, Iowa, to Chicago, Ill.

Drs. Marshall, of Philadelphia, Pa., William H. Ridge, of Trevose, Pa., and Charles F. Oat, of West Chester, Pa., officiated as veterinarians at the Philadelphia Open-air Horseshow in May.

Dr. G. E. Fetter, of Hopewell, N. J., was a visitor to the JOURNAL office in the latter part of April.

Dr. D. B. Fitzpatrick has returned to Philadelphia and re-entered upon veterinary practice in the western part of the city.

Dr. J. C. Burneson, veterinarian of the Ohio Agricultural Station, has resigned to again enter upon civil practice.

State Veterinarian Pearson, of Pennsylvania, was a visitor to Baltimore, Md., in April.

Dr. A. P. Ruhl, of Fairmount, West Virginia, is Professor of Veterinary Science in the West Virginia University at Morgantown.

Dr. H. J. Weicksel, of Shamokin, Pa., is the owner of the famous trotting horse "Paxinos," with a mark of 2.14¾.

Drs. Roe and Stone, graduates of the Chicago Veterinary College, located at Milwaukee, Wis., conduct a special kennel department in connection with their South Side Hospital.

Dr. M. W. Drake, of Philadelphia, Pa., is a member of the "Gentlemen's Driving Club" of the "Quaker City."

Dr. Richard P. Lyman, of Hartford, Conn., will, on and after May 1st, be associated with the practice of Messrs. Lyman and Osgood, at 50 Village Street, Boston, Mass.

Dr. W. H. Yingst, of Harrisburg, Pa., made two trips to South Africa for the English Government. On one voyage the loss of animals only reached four and the other reached six—a very small percentage, indeed.

Dr. Levi Johnson, of Portland, Ore., designates his hospital under the name of the "Oregon Veterinary Hospital."

Dr. William P. Fink, of Atlantic City, N. J., conducts a large farm close to this great seaside resort in connection with his city practice.

Dr. E. L. Cornman, of York, Pa., was recently called to defend an operation of docking, which was dismissed by the alderman on the evidence of its being a necessary operation.

Dr. W. P. Hanifan, of New York City, is very deeply interested in the success of the Metropolitan Truckmen's Association.

Dr. George B. Gilmore, of Pittsburg, Pa., has added a boarding stable in connection with his practice.

Dr. William A. Gross, of Brooklyn, N. Y., is also the proprietor of the Ross Street Hay Market in connection with his practice.

Dr. B. F. Bachman, of Pittsburg, Pa., has become joint owner of the Schenley Riding Academy.

Dr. H. D. Gill, of New York City's Driving Club, takes an active part in the welfare of the club, and fills well a place on several important committees.

Dr. E. G. Gilbert, of Pottstown, Pa., is the owner of a fast roadster which is high classed in her breeding.

Dr. G. A. Johnson, of Sioux City, Iowa, is the veterinary editor of the *Farm, Stock and Fireside Journal.*

Dr. John G. Miller, of Reading, Pa., is conducting a veterinary hospital in connection with his practice. Lameness and gaiting of horses are especially attractive features of veterinary science to Dr. Miller.

Dr. Charles L. Colton, of Englewood, N. J., graduate of the Veterinary Department of the University of Pennsylvania, has succeeded to the practice of Dr. R. P. Lyman, of Hartford, Conn.

Dr. M. D. Harper, of Breningsville, Pa., graduate of the New York College of Veterinary Surgeons, was a visitor to the JOURNAL office in May.

Dr. A. W. Gorham, formerly located at Port Chester, N. Y., is now at Westwood, Mass.

Dr. V. B. Weaver, of Bangor, Pa., was a visitor to the JOURNAL office in May.

Dr. James T. Glennon fills the rôle of veterinarian to the fire department of Newark, N. J.

Dr. H. B. McClellan, of Bryn Mawr, Pa., who for several years devoted his time to human medicine in one of the Western States, has returned to his home and re-entered upon the practice of veterinary medicine.

Dr. A. M. Humphrey, of Darien Centre, N. Y., has not been able to practice for two years, owing to illness, but continues to take a lively interest in veterinary matters, and is a constant reader of the JOURNAL.

Dr. G. E. Nesom, of Clemson College, S. C., will favor the JOURNAL with a report of experiences in the exhibiting of Northern cattle at the Charleston Exposition.

Dr. T. Earle Budd, of Orange, N. J., will officiate as veterinarian to the Atlantic City Horseshow, July 15th to 19th.

